# Association of change in muscle mass assessed by D_3_‐creatine dilution with changes in grip strength and walking speed

**DOI:** 10.1002/jcsm.12494

**Published:** 2019-10-17

**Authors:** Kate A. Duchowny, Katherine E. Peters, Steven R. Cummings, Eric S. Orwoll, Andrew R. Hoffman, Kristine E. Ensrud, Jane A. Cauley, William J. Evans, Peggy M. Cawthon, for the Osteoporotic Fractures in Men (MrOS) Study Research Group

**Affiliations:** ^1^ Department of Epidemiology and Biostatistics University of California, San Francisco San Francisco CA USA; ^2^ California Pacific Medical Center Research Institute University of California, San Francisco San Francisco Coordinating Center San Francisco CA USA; ^3^ Department of Medicine Oregon Health and Science University Portland OR USA; ^4^ Department of Medicine Stanford University School of Medicine Stanford CA USA; ^5^ Center for Chronic Disease Outcomes Research Minneapolis VA Health Care System Minneapolis MN USA; ^6^ Division of Epidemiology and Community Health University of Minnesota Minneapolis MN USA; ^7^ Department of Medicine University of Minnesota Minneapolis MN USA; ^8^ University of Pittsburgh Pittsburgh PA USA; ^9^ Department of Nutritional Sciences and Toxicology University of California, Berkeley Berkeley CA USA; ^10^ Division of Geriatrics Duke University Medical Center Durham NC USA

**Keywords:** Sarcopenia, Muscle mass, Grip strength, Walking speed

## Abstract

**Background:**

Muscle mass declines with age. However, common assessments used to quantify muscle mass are indirect. The D_3_‐creatine (D_3_Cr) dilution method is a direct assessment of muscle mass; however, longitudinal changes have not been examined in relation to changes in other measures of muscle mass, strength, and performance.

**Methods:**

A convenience sample of 40 men from the Osteoporotic Fractures in Men Study (mean age = 83.3 years, standard deviation = 3.9) underwent repeat assessment of D_3_Cr muscle mass, dual‐energy X‐ray absorptiometry (DXA) lean mass, grip strength, and walking speed at two time points approximately 1.6 years apart (2014–2016). One‐sample *t*‐tests and Pearson correlations were used to examine changes in DXA total body lean mass, DXA appendicular lean mass/height^2^, DXA appendicular lean mass/weight, D_3_Cr muscle mass, D_3_Cr muscle mass/weight, grip strength, walking speed, and weight.

**Results:**

D_3_‐creatine muscle mass, D_3_Cr muscle mass/weight, grip strength, and walking speed all significantly declined (all *P* < 0.01). The change in DXA measures of lean mass was moderately correlated with changes in D_3_Cr muscle mass. There was no significant correlation between the change in DXA measures of lean mass and change in walking speed (all *P* > 0.05). The change in D_3_Cr muscle mass/weight was moderately correlated with change in walking speed (*r* = 0.33, *P* < .05). The change in grip strength was weakly correlated with the change in DXA measures of lean mass and D_3_Cr muscle mass (*r* = 0.19–0.32).

**Conclusions:**

The results of our study provide new insights regarding the decline in muscle strength and D_3_Cr muscle mass. The D_3_Cr method may be a feasible tool to measure declines in muscle mass over time.

## Introduction

Researchers have postulated that the age‐related loss of muscle mass has detrimental consequences on functional capacity and risk of disability.^1^, ^2^, ^3^ Lean body mass, frequently measured using dual‐energy X‐ray absorptiometry (DXA), has been used as a surrogate measure of muscle mass. However, lean body mass has variable associations with adverse health outcomes in older adults. Indeed, several studies have found that individuals with low muscle mass as measured by DXA were at greater risk of falls and hospitalization while others have found null associations.^4^, ^5^, ^6^

One potential reason for these inconsistent findings may stem from measurement issues in how muscle mass is assessed. DXA, a commonly used approximation of muscle mass, has been shown to be both an imprecise and indirect measure of muscle mass.^7^ DXA utilizes a compartment model in which bone mass and fat mass are determined directly and are subtracted from total body mass to yield lean mass. As a result, DXA lean mass includes not only muscle mass but also water, fibrous tissue, and other non‐fat, non‐bone material (i.e. organs and tissue). We posit that a major reason for the inconsistent associations between DXA lean mass and adverse health outcomes in older adults may be due to the measurement error in the approximation of muscle mass.

The D_3_‐creatine (D_3_Cr) dilution method is a direct assessment of muscle mass.^8^, ^9^, ^10^ We found that low D_3_Cr muscle mass is strongly related to poor physical performance and subsequent falls and mobility problems in older men, but these relationships were not observed using DXA measures of lean body mass.^9^ Although these results suggest that D_3_Cr muscle mass is more highly related to important age‐related health outcomes, the longitudinal change in D_3_Cr muscle mass has not been established. In addition, it is unknown whether changes in muscle mass measured by D_3_Cr dilution mirror changes in physical performance and strength or if they reflect similar changes in lean mass by DXA. Based on the above, the primary objectives of this study were to (i) quantify the change in D_3_Cr muscle mass over time; (ii) relate the change in D_3_Cr muscle mass to concurrent changes in strength and performance; and (iii) compare and contrast these results to the changes observed in DXA lean mass.

## Methods

### Osteoporotic Fractures in Men cohort

In 2000–2002, 5994 ambulatory community‐dwelling men aged ≥65 years without bilateral hip replacements were enrolled in Osteoporotic Fractures in Men Study, a multicentre cohort study of aging and osteoporosis.^11^, ^12^ All men provided written informed consent, and the study was approved by the institutional review board at each centre.

### Study sample

In 2014–2016, 2786 survivors participated in at least some part of the ‘Visit 4' (Year 14) clinic visit that included whole‐body DXA, D_3_Cr muscle mass, and objective assessment of physical performance and strength. A volunteer convenience sample of 41 men from the Portland study site returned to the clinical centre to repeat these measurements an average of 1.6 years after the Year 14 visit. Analyses were restricted to men who had D_3_Cr muscle mass, DXA lean mass, walking speed, and grip strength measurements at both time points. Identical clinical protocols were used for all measures for both visits. One study participant who had a D_3_Cr muscle gain value greater than 30% [+3 standard deviations (SDs)] was considered an outlier and excluded, yielding a final analytic sample of *n* = 40.

### D_3_‐creatine dilution method to measure muscle mass

Muscle mass was determined by the D_3_Cr dilution method as previously described.^8^ Briefly, total body creatinine pool size, and subsequently total body muscle mass, was assessed using a single oral dose of deuterated creatine (D_3_‐creatine), which is absorbed and diluted by entry into the endogenous pool of creatine in skeletal muscle. Labelled creatinine (D_3_‐creatinine) and unlabelled creatinine are then measured in a fasting, single‐void urine sample 3–6 days after ingestion of the dose. These measures are then included in an algorithm to determine total body creatine pool size and skeletal muscle mass (kilograms).^8^ To account for body size differences across participants, the primary analyses utilized the ratio of muscle mass (kilograms) to body mass (kilograms), that is, D_3_Cr muscle mass/weight. Weight was measured using a digital scale.

### Appendicular lean mass by dual‐energy X‐ray absorptiometry

Appendicular lean mass (ALM) and body fat were assessed by whole‐body DXA scans (Hologic 4500 scanners, Waltham, MA) as previously described.^13^ ALM was standardized by weight (weight) and height^2^ because previous definitions of sarcopenia have relied on this definition.

### Muscle strength and walking speed

Grip strength (kilograms) from two tests of each hand was assessed using Jamar handheld dynamometers; the maximum value obtained across all tests was analysed. Walking speed at usual pace was measured over a 6 m course using the average of two trials (metres per second).^9^

### Physical activity

Physical activity was assessed by self‐report using the Physical Activity Scale for the Elderly questionnaire that measures the intensity, frequency, and duration of a variety of activities over a period of 7 days. Physical Activity Scale for the Elderly is a unit‐less scale based on a weighted average of responses to questions about volitional and occupational activity; higher scores indicate greater activity.^14^

### Statistical approach

One‐sample *t*‐tests were used to determine whether the changes in DXA total body lean mass, ALM/height^2^, ALM/weight, D_3_Cr muscle mass, D_3_Cr muscle mass/weight, grip strength, walking speed, and height and weight were significantly different from zero across the 1.6 years of follow‐up. Pearson correlation coefficients were calculated between these change variables. We assessed both simple linear change and per cent change. Significance was assessed with a two‐tailed alpha of 0.05. All statistical analyses were conducted using SAS software 9.4 (Cary, NC).

## Results

Study participant characteristics are presented in *Table* 1. Overall participants were older (mean age = 83.3 years, SD = 3.9) and were overweight (mean body mass index = 26.4 kg/m^2^, SD = 3.1)

**Table 1:** Participant characteristics at baseline (2006) in the Osteoporotic Fractures in Men Study (*n* = 40)

Characteristic	Mean (SD), *N* (%)
Age (years)	83.4 (3.9)
DXA total body lean mass (kg)	55.22 (4.98)
Appendicular lean mass (ALM) (kg)	23.3 (2.6)
ALM/weight	0.29 (0.03)
ALM/height^2^	7.71 (0.8)
D_3_Cr muscle mass (kg)	24.64 (3.92)
D_3_Cr muscle mass/weight	0.31 (0.04)
Height (cm)	173.9 (6)
Weight (kg)	79.7 (9.36)
Body mass index (kg/m^2^)	26.4 (3.1)
Total fat (%)	25.5 (5.6)
Maximum grip strength (kg)	37.7 (9.2)
Walking speed (m/s)	1.29 (0.22)
PASE score	129.7 (62.9)
White	35 (87.5)
Co‐morbidities	
0	15 (37.5)
1	19 (47.5)
2+	6 (15)
Smoking status	
Current smoker	0
Former smoker	20 (50)
Never smoker	18 (45)

D_3_Cr, D_3_‐creatine; DXA, dual‐energy X‐ray absorptiometry; PASE, Physical Activity Scale for the Elderly; SD, standard deviation.

**Figure 1:**
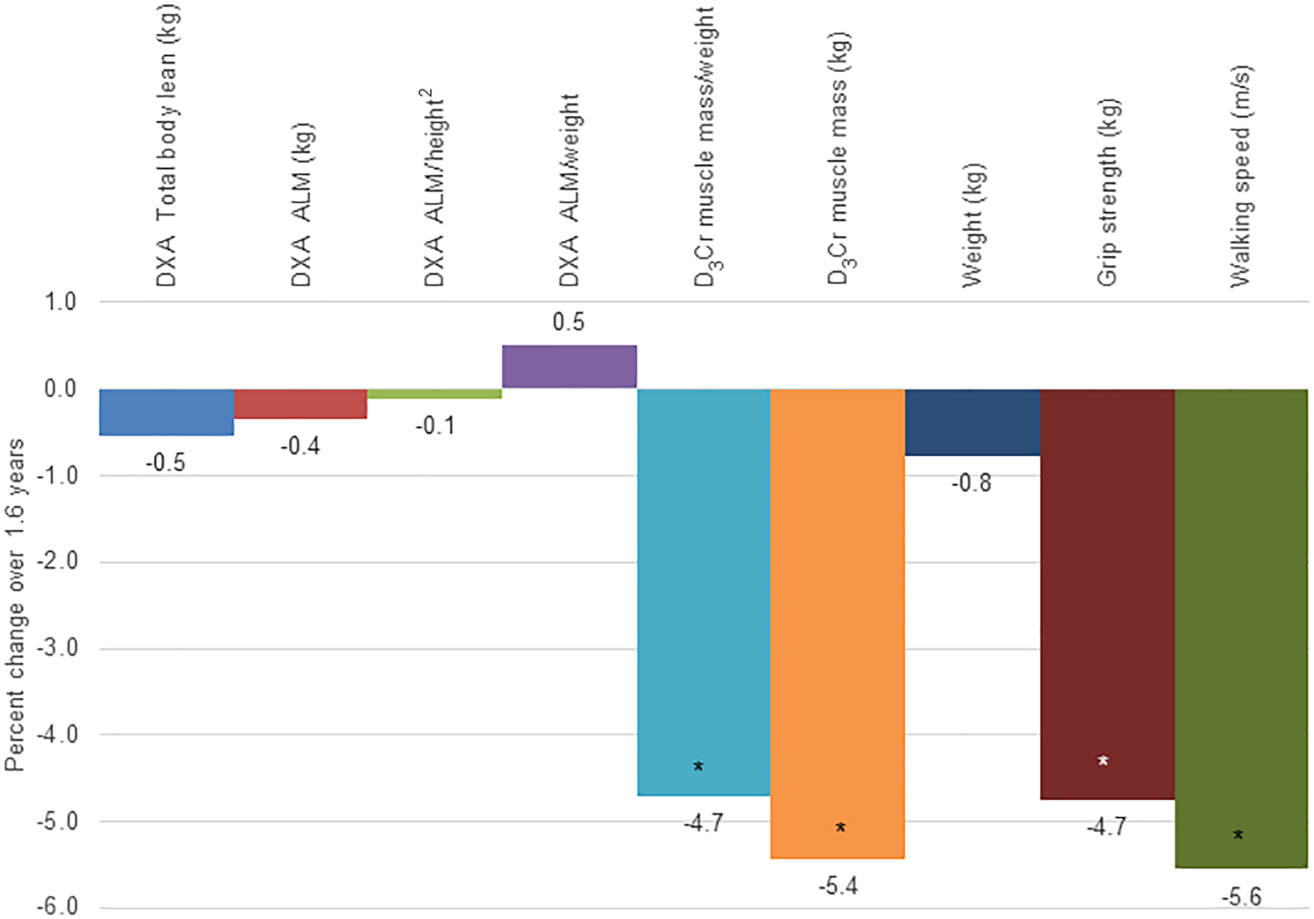
Per cent change in dual‐energy X‐ray absorptiometry (DXA) lean mass and appendicular lean mass (ALM), D_3_‐creatine (D_3_Cr) muscle mass, grip strength, and walking speed across 1.6 years (*n* = 40).^*^ *P* < 0.01 for per cent change significantly different from zero.

Across an average of 1.6 years, D_3_Cr muscle mass, D_3_Cr muscle mass/weight, grip strength, and walking speed all significantly declined (*P* < 0.01 for all) (*Table* 2). The decline for D_3_Cr muscle mass, D_3_Cr muscle mass/weight, grip strength, and walking speed ranged from 4% to 5% (*Figure* 1). In contrast, there were no significant simple linear changes or per cent changes in DXA total body lean mass, DXA ALM, DXA ALM/height^2^, or DXA ALM/weight (*P* > 0.05 for all).

**Table 2:** Simple linear change in DXA lean mass and ALM, D_3_Cr muscle mass, grip strength, and walking speed across 1.6 years (*n* = 40)

Variable	Simple linear change				
	Mean	Minimum	Maximum	SD	*P* *
DXA total body lean mass (kg)	−0.31	−3.61	2.99	1.57	0.217
DXA ALM (kg)	−0.08	−1.80	1.65	0.83	0.552
DXA ALM/height^2^	−0.01	−0.61	0.57	0.28	0.803
DXA ALM/weight	0.00	−0.03	0.03	0.01	0.376
D_3_Cr muscle mass/weight	−0.02	−0.07	0.03	0.02	<0.0001
D_3_Cr muscle mass (kg)	−1.42	−5.75	2.47	1.84	<0.0001
Weight (kg)	−0.63	−6.70	6.70	2.92	0.179
Grip strength (kg)	−1.95	−12.00	10.00	4.19	0.005
Walking speed (m/s)	−0.08	−0.45	0.37	0.18	0.006
PASE activity	−2.54	−124.10	97.50	46.42	0.731

ALM, appendicular lean mass; D_3_Cr, D_3_‐creatine; DXA, dual‐energy X‐ray absorptiometry; PASE, Physical Activity Scale for the Elderly; SD, standard deviation.

* Test if mean change significantly different from zero.

The correlations between the per cent changes in both D3Cr muscle mass and DXA lean mass are presented in *Table* 3. The change in D_3_Cr muscle mass was moderately correlated with the change in total body lean mass as measured by DXA (*r* = 0.50, *P* < .001). The correlation between the change in D_3_Cr muscle mass/weight was not significantly associated with the change in DXA total body lean mass (*r* = 0.24). The change in DXA ALM, DXA ALM/height^2^, and DXA ALM/weight was modestly correlated with the change in D_3_Cr muscle mass/weight (*r* = 0.36–0.46). However, while the change in DXA ALM and DXA ALM/height^2^ was more strongly correlated to the change in D_3_Cr muscle mass, the change in DXA ALM/weight was not significantly correlated with the change in D_3_Cr muscle mass.

**Table 3:** Correlation between the per cent change in DXA lean mass, ALM, and D_3_Cr muscle mass across 1.6 years (*n* = 40)

Pearson correlation coefficient *P*‐value	Δ D_3_Cr muscle mass/weight	Δ D_3_Cr muscle mass
Δ DXA total body lean mass	**0.24**	**0.50**
	*0.136*	*<0.001*
Δ DXA ALM	**0.44**	**0.58**
	*0.004*	*<0.001*
Δ DXA ALM/height^2^	**0.46**	**0.57**
	*0.003*	*<0.001*
Δ DXA ALM/weight	**0.36**	**0.03**
	*0.024*	*0.873*

*Note*: Bold values = r, correlation; Italic value = corresponding p‐value.

ALM, appendicular lean mass; D_3_Cr, D_3_‐creatine; DXA, dual‐energy X‐ray absorptiometry.

There was no significant correlation between the change in DXA measures of lean mass (total body, ALM/height^2^, and ALM/weight) and change in walking speed. In contrast, the change in D_3_Cr muscle mass/weight was moderately correlated with change in walking speed (*r* = 0.33); the correlation between the change in D_3_Cr muscle mass and change in walking speed was of borderline significance (*r* = 0.29) (*Table* 4).

**Table 4:** Correlation between the per cent change in DXA lean mass, ALM, and D_3_Cr muscle mass and changes in grip strength and walking speed across 1.6 years (*n* = 40)

	DXA lean mass				D_3_Cr muscle mass	
Pearson correlation coefficient *P*‐value	Δ DXA total body lean mass	Δ ALM	Δ ALM/height^2^	Δ ALM/weight	Δ D_3_Cr muscle mass	Δ D_3_Cr muscle mass/weight
Δ Grip strength	**0.32**	**0.29**	**0.26**	**0.19**	**0.20**	**0.19**
	*0.042*	*0.067*	*0.109*	*0.251*	*0.209*	*0.241*
Δ Walking speed	**−0.11**	**−0.09**	**−0.08**	**−0.10**	**0.29**	**0.33**
	*0.513*	*0.573*	*0.604*	*0.555*	*0.069*	*0.038*

*Note*: Bold values = r, correlation; Italic value = corresponding p‐value.

ALM, appendicular lean mass; D_3_Cr, D_3_‐creatine; DXA, dual‐energy X‐ray absorptiometry.

**Figure 2:**
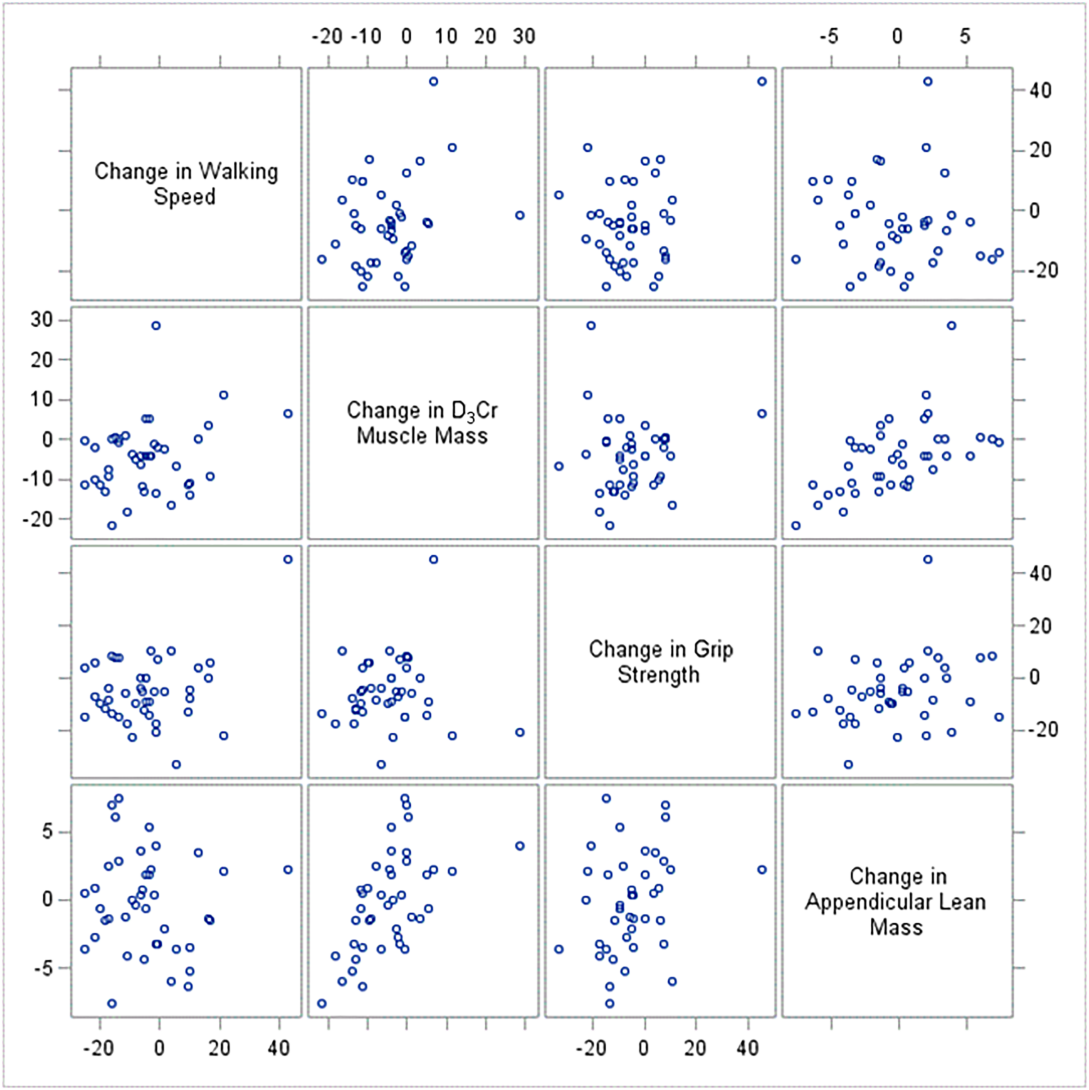
Scatter plot for association between per cent change in walking speed, D_3_‐creatine muscle mass, grip strength, and dual‐energy X‐ray absorptiometry appendicular lean mass.

The correlation between the change in grip strength and change in DXA lean mass or D_3_Cr muscle mass was weak (0.19–0.32); only the correlation between the change in grip strength and the change in DXA total body lean mass reached statistical significance (*P* = 0.04). The correlations between the change in ALM, D_3_Cr muscle mass, grip strength, and walking speed are presented in Figure 2.

## Discussion

In our convenience sample of older men, we showed that skeletal muscle mass rapidly decreases with aging and this is detected more accurately by a stable isotope dilution method of measuring mass than by DXA. Overall, we found that D_3_Cr muscle mass declined over 1.6 years and this change was correlated with concurrent declines in walking speed, but not with declines in grip strength. In contrast, we did not observe a similar pattern of change with DXA measures of lean mass across the same time period, nor was there a significant relationship between the change in DXA measures of lean mass and declines in walking speed. The magnitude of per cent change was similar for D_3_Cr muscle mass, grip strength, and walking speed while the change in DXA lean mass was negligible. The change in D_3_Cr muscle mass was modestly related to the change in grip strength, although this finding was not statistically significant. DXA lean mass had a modest association with the change in grip strength, even after accounting for body mass. Taken together, our results suggest that changes in D_3_Cr muscle mass are more closely related to changes in performance than the changes in DXA measures of lean mass, while the relation between changes in grip strength and DXA or D_3_Cr‐based measures of muscle mass appear more similar.

There is much debate as to whether muscle strength and muscle mass decline at the same rate over time.^15^, ^16^ Previous studies have suggested a disconnect between declines in approximations of muscle mass with changes in strength, with mixed evidence as to whether one outpaces the other.^4^, ^17^, ^18^ For example, in the Health ABC Study, older Black and White men and women had a three‐fold greater loss in strength than the loss in DXA lean mass across 3 years.^16^ In a randomized controlled trial examining the efficacy of diet and exercise among 73 older persons with osteoarthritis, significant declines in DXA lean mass were observed while muscle strength increased among the intervention group.^19^

Our study provides new insight into the relationship between muscle strength and D_3_Cr muscle mass by showing that changes in performance may be more related to declines in muscle mass than previously thought. Further, we posit that the apparent discrepancy between our results and previous studies may have been driven by imprecise measurements of muscle mass. While the D_3_Cr method may have higher variability than highly precise DXA measures, our data suggest that the D_3_Cr dilution method may provide greater accuracy in measuring and detecting changes in muscle mass. This is supported by prior work showing that D_3_Cr muscle mass is strongly related to physical performance, functional status, and incident injurious falls and mobility limitations, whereas DXA lean mass is not associated with these outcomes.^9^ Taken together, the D_3_Cr method can provide a useful and more accurate assessment of muscle mass in its ability to both quantify declines over time while also identifying those who may be most at risk for future negative health outcomes on account of lower muscle mass.

Several limitations must be noted. First, the small sample size may have hindered our ability to meaningfully detect large effect sizes. For example, several of the correlations reported were in the low to moderate range and were of borderline significance. A larger sample size would enable more precise quantification of these correlations while also providing the opportunity to run linear regression models with adjustment for potential confounders. Second, the men who were included in this study were very old and mostly White. As a result, our findings have limited generalizability to other groups, including women, which is particularly important given known gender differences in body size and composition.^20^ Future studies should include women, the younger old, and other groups as a way expanding the external validity of the results presented in this study. Third, the convenience sample of men in our study was likely healthier than those not included in the analysis. Those not included in our analyses may have had greater declines in muscle mass, strength, and performance, and we may have underestimated this change. Lastly, this study was conducted across a short follow‐up window. Changes over longer periods or across more time points may reflect different patterns and alter our conclusions.

## Conclusions

This is the first study to examine how change in muscle mass, as assessed by D_3_Cr dilution, is linked to changes in hand grip strength and walking speed. Unlike previous studies that have largely relied on DXA to quantify changes in the approximation of lean mass, we demonstrated that D_3_Cr muscle mass may be a more appropriate indicator of muscle mass and is capable of assessing declines in muscle mass over time, which to date, has been equivocal. These results suggest a substantial and, previously unreported, loss of skeletal muscle mass over a relatively short period of time in this population of very old men. Our results also suggest that the interrelationship between the change in muscle mass with concurrent changes in strength and physical performance may have been underestimated. However, future research to characterize these changes is needed. This study sheds light on the importance of using a novel and accurate measure of muscle mass over time and lays the groundwork for future research that seeks to quantify the link between *muscle mass* and adverse health outcomes in a longitudinal setting.

## Funding

The Osteoporotic Fractures in Men Study is supported by National Institutes of Health funding. The following institutes provided support: the National Institute on Aging (NIA), the National Institute of Arthritis and Musculoskeletal and Skin Diseases (NIAMS), the National Center for Advancing Translational Sciences (NCATS), and NIH Roadmap for Medical Research under the following grant numbers: U01 AG027810, U01 AG042124, U01 AG042139, U01 AG042140, U01 AG042143, U01AG042145, U01 AG042168, U01 AR066160, and UL1 TR000128. Funding for the D_3_Cr muscle mass measure was provided by NIAMS (grant number R01 AR065268). GlaxoSmithKline provided in‐kind support by providing the D_3_‐creatine dose and analysis of urine samples.This research was also supported by the National Institute on Aging of the National Institutes of Health under Award T32‐AG049663.

## Author contributions

K.A.D. and P.M.C. drafted initial version of the manuscript; K.E.P. and K.A.D. completed statistical analyses; S.R.C., E.S.O., A.R.F., K.E.E., J.A.C., and P.M.C. secured funding for the acquisition of data; all authors provided critical review of the manuscript.

## Conflict of interest

K.A.D., K.E.P., S.R.C., E.S.O., A.R.H., K.E.E., J.A.C., W.J.E., and P.M.C. have no conflicts of interest to disclose.

## Sponsor's role

The funders had no role in the design and conduct of the study; the collection, analysis, and interpretation of the data; or the preparation, review, or approval of the manuscript.
